# Stable rat models in constipation with depression and anxiety: evaluation and validation

**DOI:** 10.3389/fnbeh.2026.1745331

**Published:** 2026-04-09

**Authors:** Jiali Liu, Shaoliang Li, Conghui Bian, Hongxia Mi, Tiantian He, Haihua Qian, Dan Zhang

**Affiliations:** 1 Department of Colorectal Surgery, The Second Affiliated Hospital of Guangzhou University of Chinese Medicine, the Second Clinical Medical College of Guangzhou University of Chinese Medicine, Guangzhou, China; 2 Department of Anorectal Surgery, Affiliated Hospital of Nanjing University of Chinese Medicine, Nanjing, China

**Keywords:** animal models, apoptosis proteins, CUMS, FC with anxiety and depression, hippocampus

## Abstract

**Purpose:**

The study aimed to establish a model of functional constipation (FC) with anxiety and depression in rats using the compound diphenoxylate combined with chronic unpredictable mild stress (CUMS). It further investigated constipation and behavioral indicators in different groups of various compounds of diphenoxylate with CUMS durations to determine the optimal time frame for successful model establishment.

**Method:**

Thirty-two female rats were randomly assigned to control, FC before CUMS, FC after CUMS, and FC throughout CUMS groups. Control: Same amount of regular saline solution, FC before CUMS: Compound diphenoxylate 15 mg/kg 2 weeks + CUMS 5 weeks, FC after CUMS: CUMS 5 weeks + Compound diphenoxylate 15 mg/kg 2 weeks, FC throughout CUMS: CUMS 5 weeks with compound diphenoxylate 10 mg/kg for 4 weeks. After model establishment, constipation and behavioral indicators were detected. HE staining was used to observe pathological changes in the brain and colon tissue, while AB-PAS was used to observe goblet cells in the colon tissue, and Nissl staining was used to observe neuron cells in the hippocampus. The protein expression of BDNF and Bax in the hippocampus and PGP9.5 and iNOS in the colon was detected by WB.

**Results:**

In the FC throughout group, synaptic ultrastructure significantly decreased, aligning with pathological changes, indicators of FC with anxiety and depression. The protein expression levels of BDNF and PGP9.5 were markedly decreased, and the levels of Bax and iNOS were elevated. This study provided a reliable reference for determining the optimal establishment way of FC with anxiety and depression models and for *in vivo* FC with anxiety and depression animal experiments.

## Graphical Abstract

**Figure:**
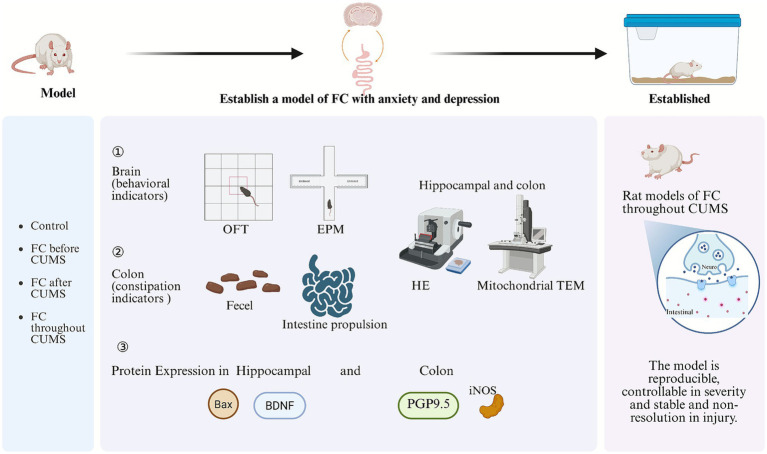

## Introduction

Functional constipation (FC) is a prevalent gastrointestinal disease whose incidence rates are on the rise year after year (Palsson et al., 2020). The worldwide prevalence of functional constipation, according to all the studies, is 14% (Michael et al., 2017). Many patients show mental overload before the diagnosis of bowel disease and face a considerably higher prevalence of depression compared to the healthy population before the diagnosis (Jonathan et al., 2021). Anxiety and depression are more common among functional constipation patients than among healthy individuals (Reema et al., 2014). Therefore, effective prevention and treatment of functional constipation also need to focus on mental disorders.

Currently, there are many methods to establish a functional constipation model to observe the behavioral assessment, for example, loperamide hydrochloride, diphenoxylate, intraperitoneal loperamide, etc. However, the most commonly used method is still the constipation model, established to observe the depressive and anxious-like behaviors, and all of them have a short modeling, which is incompatible with human constipation with depression and anxiety induced in the long term. The most common model of constipation with depression is loperamide hydrochloride and diphenoxylate, and there is one study mentioning loperamide with CUMS (Xu et al., 2018; Li et al., 2024; Kong et al., 2024; Zhou et al., 2024; Zou et al., 2024; Wang et al., 2025; Wu et al., 2026; Chen et al., 2025), as shown in Table 1.

**Table 1:** FC with depression establishment in the literature.

Model	Animal	Symptoms	Date
Loperamide (5 mg/kg) was given intraperitoneally twice a day for 7 days	Mice	Significant increase in the immobility time of Forced Swimming Test (FST) and the greatest increase in time spent immobile in the Tail Suspension Test (TST)	2018 (Xu et al., 2018)
Intragastric loperamide hydrochloride (1.5 mg/kg twice daily) for 14 days	Mice	Lower preference for the Sucrose Preference Test (SPT) and a longer immobility time	2024 (Li et al., 2024)
Distilled water complemented with 20 mg/kg diphenoxylate for 14 days.	Mice	Reduced struggle time, increased buried balls, and decreased exploration of open arms	2024 (Kong et al., 2024)
Loperamide (10 mg/kg) by oral gavage twice a day for 10 days	Mice	Total distance traveled in the Open Field Test (OFT) was decreased, but the immobility time and duration were increased	2024 (Zhou et al., 2024)
Diphenoxylate added in water (20 mg/kg) 14 days	Mice	The Elevated Plus Maze test (EPM) results showed that increasing the marbles buried counts	2024 (Zou et al., 2024)
Diphenoxylate orally 14 days	Mice	More buried marbles in the Marble buried test; longer immobility time in TST	2025 (Wang et al., 2025)
Loperamide in sterile water (0.8 mg/kg) twice daily for 10 days.; after constipation induction, CUMS on 28 days	Mice	In the SPT, significantly reduced sucrose preference, OFT exhibited longer resting times, reduced activity duration, and shorter total movement distances.; TST and FST, showed prolonged immobility durations	2025 (Wu et al., 2026)
CUMS 6 weeks	Mice	Crossed the platform fewer times, lower sucrose preference, reduction in both the total movement distance moved and the time spent in the central area	2025 (Chen et al., 2025)

This experiment was mainly intended to explore the model of constipation with depression and anxiety. A previous study reported increased incidence of depression and anxiety in female mice, following chronic mild stress exposure, suggesting that, unlike males, females were more susceptible to 5-HT depletion and its mood-depressing effects (Sachs et al., 2014). Thus, female rats were an appropriate option to observe disorders that were reportedly prevalent in women, such as IBS and depression (Radu et al., 2020). Current studies establish FC with depression through loperamide in the constipation model, after constipation-induced CUMS on the 28th day (Wu et al., 2026). At present, most scholars focus on the constipation model in behavioral indicator, while the model study of FC with depression and anxiety has been less reported. There were many animal models of depression that used CUMS as a model. In CUMS animal model, neurogenesis was impaired, and disorders were more readily manifested as a result of physical and psychological stress (Coe et al., 2003; Mirescu et al., 2004). Diphenoxylate, as a constipation model medicine, has direct effects on intestinal smooth muscle and inhibits peristalsis as a result of delaying intestinal contents of advancing speed and reducing bowel movements (Xichuan et al., 2018; Baldassano et al., 2010). Diphenoxylate is an example of opiates that only crosses the blood–brain barrier (BBB) to a very limited degree, thereby having much greater action in the systemic environment, and hence is useful as an antidiarrheal agent without the problems associated with central nervous system effects (Dehaven-Hudkins et al., 1999; Crowe and Wong, 2003).

Therefore, in this experiment, female rats were used to investigate the compound diphenoxylate with CUMS modeling time of (FC before CUMS, FC after CUMS, and FC throughout CUMS) constipation and behavioral, pathological indicators, transmission electron microscopy, and proteins related to cholesterol-reversal pathways at different modeling. It can provide a reliable reference for the establishment of an FC with a depression and anxiety model. It may be possible to pinpoint and clarify the mechanism involved with FC by studying animal models, allowing full disclosure and clarification of the effects of pathophysiological mechanisms on gut and brain tissue in patients with FC. Whereas, at present, the most common methods for experimental studies of FC are to establish constipation models to observe the depressive-like and anxious-like behaviors or to establish depression models to observe the constipation symptoms. The contributions of this research are summarized as follows:

1. Existing animal models of FC with anxiety and depression are inadequate for reproducing all the pathophysiological long-lasting features of human FC with anxiety and depression. This study established the exhibiting concurrent and persistently maintained symptoms of constipation alongside anxiety and depression. This model more closely mirrored the clinical presentation of patients experiencing chronic constipation accompanied by anxiety and depressive symptoms.
2. Histopathological and transmission electron microscopy were employed to determine the presence of brain and colon damage in the rats. Additionally, the structure of mitochondria in hippocampal neurons was explored to detect severity.
3. By detecting apoptosis-related proteins in brain–gut neurons, the potential mechanisms of the brain–gut axis injury would be explored.

## Materials and methods

### Animal experiments

Sprague–Dawley female rats (6 weeks of age, 200 ± 20 g) were bought from Jiangsu Huachuang Sino Laboratory Animal Technology Co., Ltd. in Taizhou, China. (Animal certificate number: SCXK (Su)-2020–0009). All rats were acclimated for 1 week under 12 h light/dark (20–25 °C) with free access to water and food. All procedures were permitted by the Animal Ethics Committee of Affiliated Hospital of Nanjing University of Chinese Medicine (approval number 2023DE-045-02).

### Rat model for FC with depression

Eight experimental rats per group were randomly distributed into the control group, FC before CUMS group, FC after CUMS group, and FC throughout CUMS group. The FC model was intragastric for compound diphenoxylate tablets, which were purchased from Jiangsu Huayang Pharmaceutical Co. Ltd. (2001035). CUMS was combined with isolation (rat cage box isolation). The CUMS process contained 11 diverse stressors, which were randomly settled throughout 35 consecutive days. The stressors were: (i) 24-h fasting; (ii) 24-h water fasting; (iii) tail clamp for 1 min; (iv) 17 h exposure to a 45° tilted cage; (v) overnight lighting; (vi) 24-h exposure to a wet cage; (vii) swimming in cold water (4 °C) for 5 min; (viii) 24-h cage disturbance; (ix) 6-h white noise stimulation (85 dB); (x) 6-h restraint in a tube; (xi) horizontal oscillation for 5 min (160 Hz) (Huang et al., 2020).

**Figure 1:**
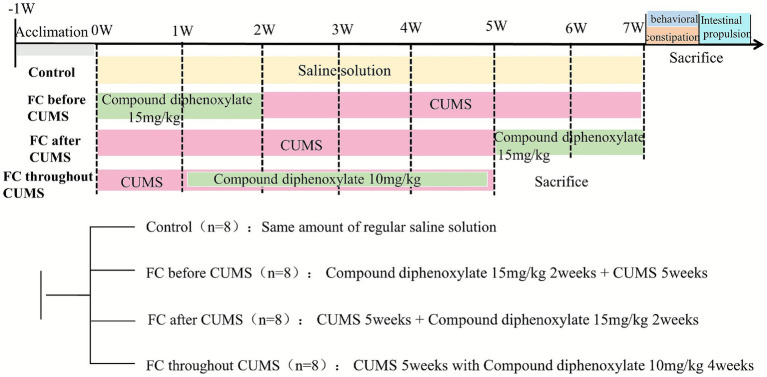
Experimental protocol of the study.

The experimental protocol of the study (Figure 1).

### Observation of rat signs

#### The body weight

The body weight of each rat was measured at 8 a.m. every Monday during the experimental period.

#### Stool parameters

The rats in every group were fasted but had free access to water for 24 h. Every rat was intragastrically administered with 3 mL of activated carbon starting from the first black defecation till time of being administered with ink. Rats were placed in metabolic cages, the total amount of 24 h feces of the rats was weighed on a balance, and their wet weights were recorded (A), then their 24h feces was placed in the setup at 110 °C constant temperature oven for 3 h. After 3 h, the dried feces were taken out, and their dry weights were recorded (B). Calculation method: the fecal moisture percentage (%) = (A-B)/A × 100%.

#### Time of first black stool

Rats were fasted for 24 h without water restriction. Each rat was given intragastric administration with 3 mL activated carbon (10% charcoal, Changtian Pharma, Heibei). The time of the first black stool was recorded starting from the end of intragastric administration.

#### Measurement of intestinal propulsion

The rats in every group were fasted but had free access to water for 24 h. Every rat was intragastrically administered 3 mL of activated carbon. After 30 min, under the inhalation of 0.5–0.7 L/min isoflurane anesthesia, the small bowels were quickly anatomized and scaled. Measurements were taken of the full length of each small bowel from the pyloric sphincter to the ileocecal valve, as well as the distance traveled by the carbon meal from the pylorus. Intestinal propulsion rate (%) = transited distance of charcoal meal/full small intestine length × 100%.

#### Behavioral assessment

SPT (Strekalova et al., 2022): For the sucrose preference test, Each rat was provided with one bottle of tap water and another bottle containing 2% sucrose solution for 5 h (19:00–24:00), and the amounts of sucrose solution and water consumed were recorded. Sucrose preference percentage (%) = sucrose consumption (mL)/(sucrose consumption (mL) + water consumption (mL)) × 100%. 2% sucrose solution was used for 48 h before the sucrose preference test was carried out, and water was deprived 12 h before the test.

### Elevated plus maze

The EPM was used to assess anxiety-like behavior of animals, which consisted of two open arms and two opposing wall-closed arms 50 cm above the ground. The time spent in different arms and the total moving distance of each rat were meticulously quantified over a 5-min intervals. Animal behavioral analysis software was used from Shanghai Jiliang Software Technology Co., Ltd.

### Open-field test

The open-field apparatus consisted of a square arena with 35 cm high walls made of black aluminum alloy board. The total distance and center time were recorded for 5 min for rats in all the groups. Animal behavioral analysis software was used from Shanghai Jiliang Software Technology Co., Ltd.

### Histopathological analysis

Two centimeters of the colon tissue, which was 2 cm away from the anus, was harvested and soaked in 4% paraformaldehyde, and then embedded in paraffin. Colon tissue of 4-μm thickness was stained with Hematoxylin–Eosin and Alcian blue stain and Periodic acid Schiff (AB-PAS). The brain tissue was made to soak with 4% paraformaldehyde, and 4 μm thickness were stained with Hematoxylin–Eosin (HE) and Nissl staining. Microscopy: to examine the stained sections under a microscope and capture images for analysis (Olympus, Tokyo, Japan).

### Transmission electron microscopy

According to the previously described method (Tan et al., 2018), fresh colonic and hippocampus tissue samples (1 mm^3^) were harvested and promptly fixed in electron microscopy fixative at 4 °C overnight, and in 1% osmium tetroxide for 2 h. They were dehydrated in graded ethanol and 100% ethanol and embedded to polymerize. Then the section was cut into 1,000 nm and 100 nm slices, stained with lead citrate and uranyl acetate, and then inspected by using transmission electron microscopy.

### Western blotting

The bicinchoninic acid (BCA) method (Beyotime, China) was used to quantify the protein. Glyceraldehyde-3-phosphate dehydrogenase (GAPDH), Bcl-2-associated X protein (Bax), brain-derived neurotrophic factor (BDNF), inducible nitric oxide synthase (iNOS), and protein gene product 9.5 (PGP9.5) in each group (Bioss Antibodies Co., Ltd.) were segregated by SDS-PAGE and transferred to polyvinylidene difluoride membranes. After blotting with 5% non-fat milk, the membrane was probed with primary antibodies, incubated with secondary antibodies, and then visualized by Gel Imaging System (RED, alpha, American), and at last, the protein expression was calculated.

### Statistical analysis

Statistical analyses were conducted using the SPSS 22.0 software. GraphPad Prism 8.0 software was used to carry out all statistical graph. With one-way analysis of variance (ANOVA) followed by Tukey’s test, remarkable differences were determined between groups, and the results were expressed as mean ± standard deviation (SD). *p*-value < 0.05 indicated a statistically significant difference.

## Results

### Difference of depression exposure periods on FC rat

#### Body weight among different groups

**Figure 2:**
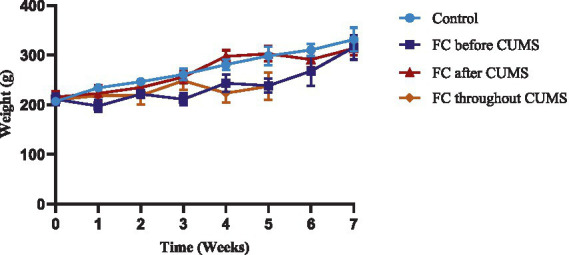
Body weight of each group.

The parameters of four groups of rats are displayed in Table 2. The initial body weight of each group was measured with no differences. After the end of modeling, the body weight gained of all four groups showed a significant decrease, which was found in the FC throughout CUMS (237 ± 27.78 g) group compared with the control group, while the weight of the FC throughout CUMS group was increased more slowly (*p* < 0.01) (Figure 2).

**Table 2:** Different group on body weight (g).

Parameters of rat	Control	FC before CUMS	FC after CUMS	FC throughout CUMS
Initial body wt (g)	206.67 ± 5.77	211.1 ± 12.16	215.33 ± 12.05	209.67 ± 5.86
Final body wt (g)	331.33 ± 24.11	315.67 ± 24.79^##^	313.33 ± 13.58^##^	237 ± 27.78^**^

Difference from the control group, ^*^p < 0.05, ^**^p < 0.01. Difference from the FC throughout CUMS group, ^#^*p* < 0.05, ^##^*p* < 0.01. Values were expressed as the mean ± SD.

#### Defecation effect of each group

**Figure 3:**
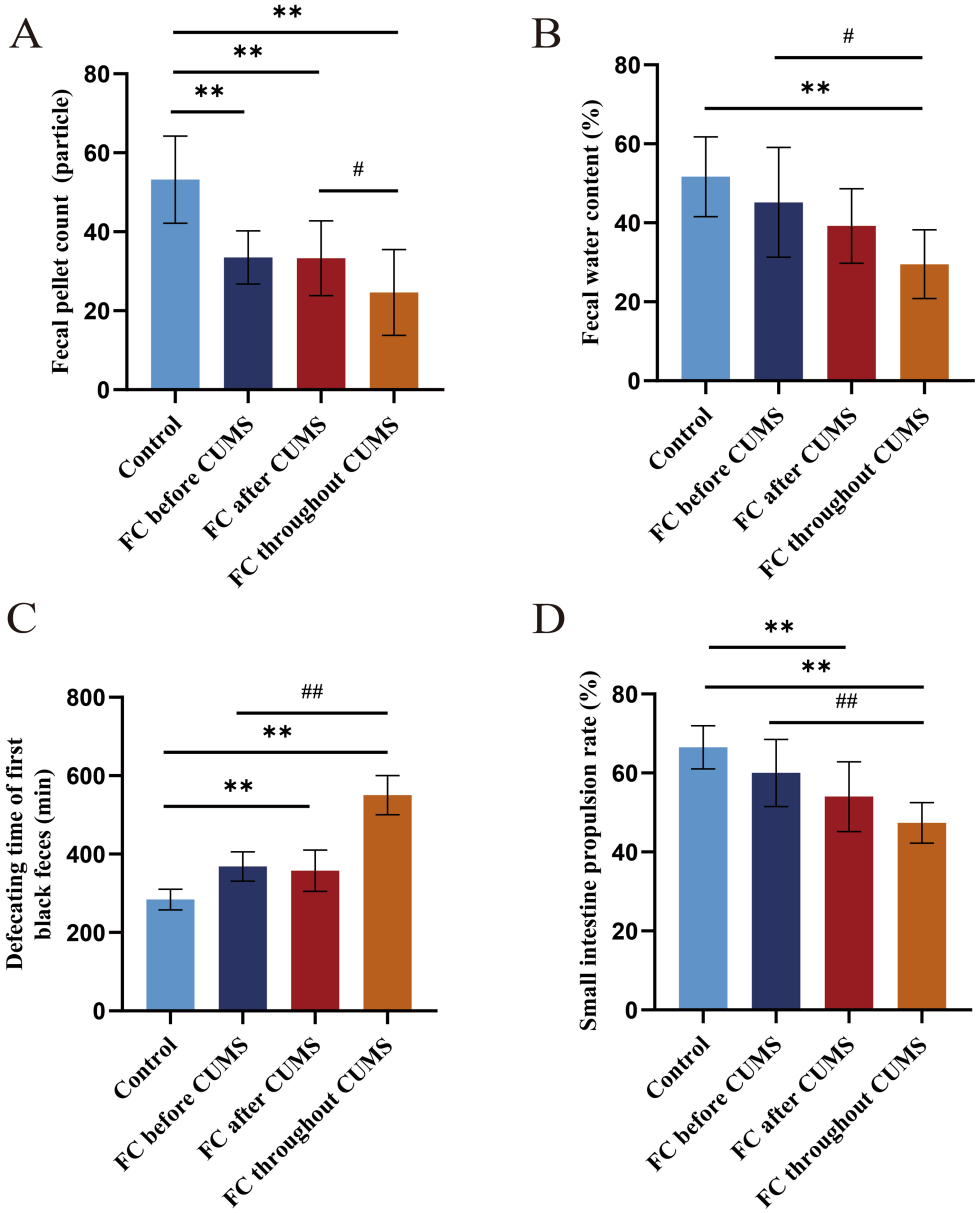
Constipation-related indicators in each group: **(A)** fecal pellet count; **(B)** fecal water content; **(C)** defecating time of first black feces; **(D)** small intestine propulsion rate. Difference from the control group, ^*^p < 0.05, ^**^p < 0.01. Difference from the FC throughout CUMS group, ^#^*p* < 0.05, ^##^*p* < 0.01. Values were expressed as the mean ± SD.

After the end of modeling, the parameters of four groups of rats were displayed in Figure 3. The fecal pellet count, fecal water content, and small intestine propulsion rate of FC throughout CUMS groups were decreased compared with the control group (*p* < 0.05). The defecating time of the first black feces of FC after CUMS and FC throughout CUMS groups was increased compared with the control group. It was found that in the FC throughout CUMS group, the defecation effect performed the worst.

#### Behavioral assessment of each group

The SPT was the most used behavioral experimental method for evaluating anhedonia. Behavioral experiments were the most direct and effective method to investigate emotional changes in animals (Zhang et al., 2023). Therefore, SPT, OFT, and EPM were used to observe the indicator of compound diphenoxylate combined with CUMS-induced depression and anxiety-like behavior.

#### OFT result

**Figure 4:**
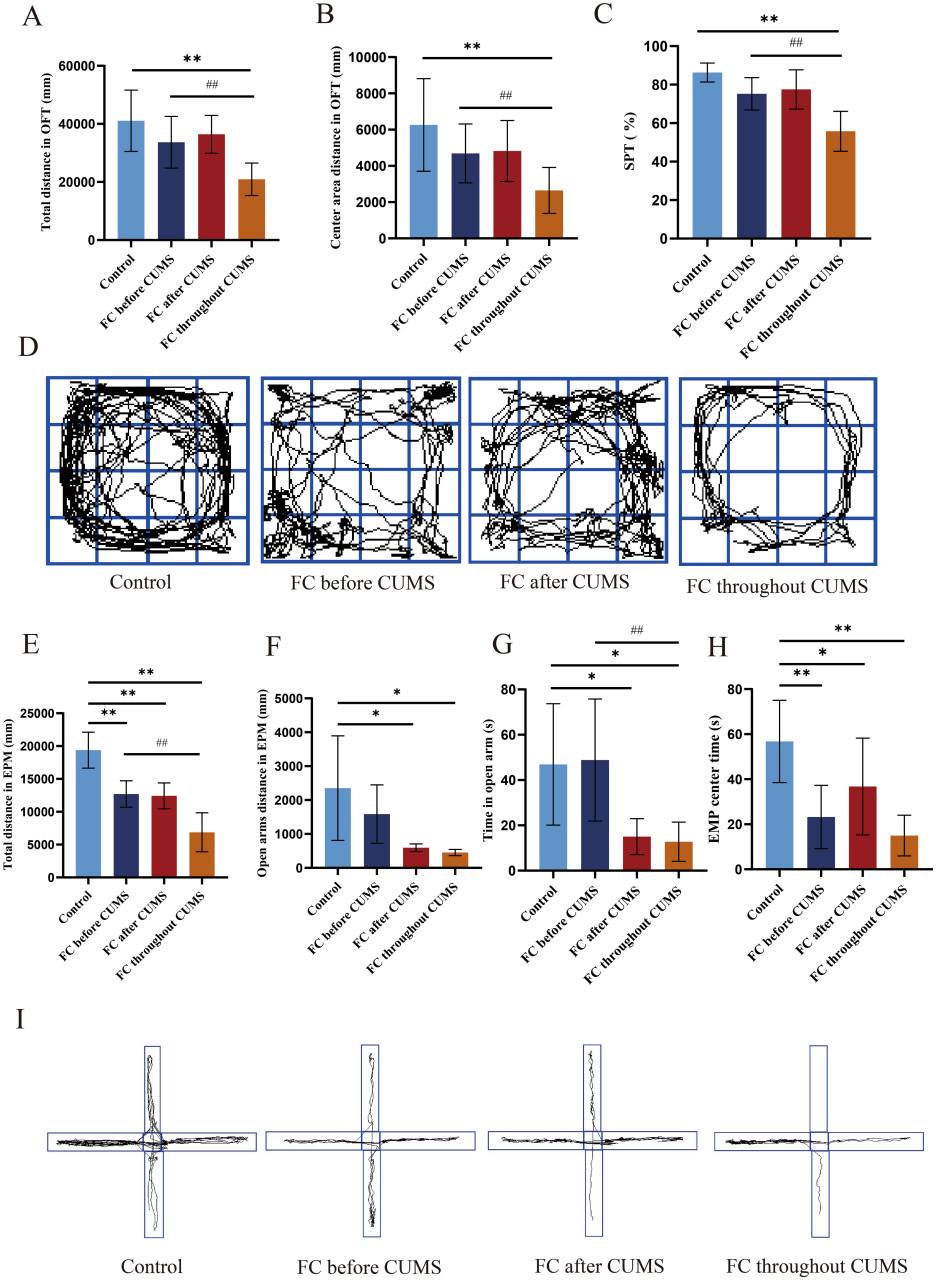
Depression-like behaviors in each group: **(A)** total distance in OFT; **(B)** center time in OFT; **(C)** sucrose preference; **(D)** representative trajectories of OFT; **(E)** total distance in EPM; **(F)** open arms distance in EPM; **(G)** time of open arm in EPM; **(H)** time of center region in EPM; **(I)** representative trajectories of EMP. Difference in the control group, *p < 0.05, **p < 0.01. Difference in the FC throughout CUMS group, ^#^*p* < 0.05, ^##^*p* < 0.01. Values are expressed as the mean ± SD.

After the end of modeling, the total distance in OFT of the three groups of rats was decreased. The total and center area distance in OFT showed a significant difference compared with the control group and FC throughout group (*p* < 0.01). Compared with the FC throughout group, the total and center area distance in OFT, FC before CUMS group showed a significant difference (*p* < 0.01) (Figures 4A,B). Compared with the control group, trajectories in other groups had decreased, especially the FC throughout group (Figure 4D).

#### SPT result

After the end of modeling, the SPT of three model groups of rats showed a significant decrease, which was observed in the FC throughout group compared with the control group (*p* < 0.01). Compared with the FC before CUMS group, the SPT of FC throughout group had significantly decreased (*p* < 0.01) (Figure 4C).

#### EPM result

The total and open arms distance moved in EPM, the time of open arm and center region was significantly shortened in the FC after CUMS and FC throughout groups compared with that in the control group (*p* < 0.05) (Figures 4E–H). Compared with the control group, trajectories in other groups had decreased, especially in the FC throughout group (Figure 4I).

#### Changes in histological alterations

**Figure 5:**
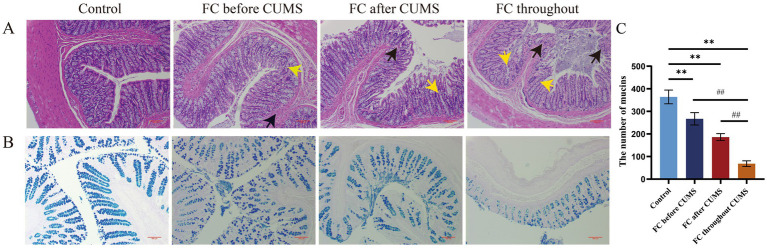
**(A)** HE staining of colon tissues in each group. Black arrow: inflammatory cells; Yellow arrow: goblet cells. **(B)** AB-PAS staining of colon tissues in each group (bars, 100 μm); difference from the control group, ^*^p < 0.05, ^**^p < 0.01. Difference from the FC throughout CUMS group, ^#^*p* < 0.05, ^##^*p* < 0.01. Values were expressed as the mean ± SD.

In the control group, abundant glands, clear structure of U-shaped crypts and cup cells, tight cell arrangement, and no inflammatory cell infiltration could be seen in the colonic tissue of rats. In the FC throughout group, the integrity of the mucosa was poor, the glands were lost, the U-shaped crypts and cup cells disappeared, and a large number of inflammatory cells infiltrated the mucous membrane with breaks in each layer. Compared with the FC throughout group, the FC before and after CUMS groups showed a reduction in mucosal edema, neatly arranged glands, and reduced inflammatory cell infiltration (Figures 5A,B). The FC throughout group demonstrated a reduced number of intestinal mucins compared to the other groups (Figure 5).

#### HE of hippocampal tissues

**Figure 6:**
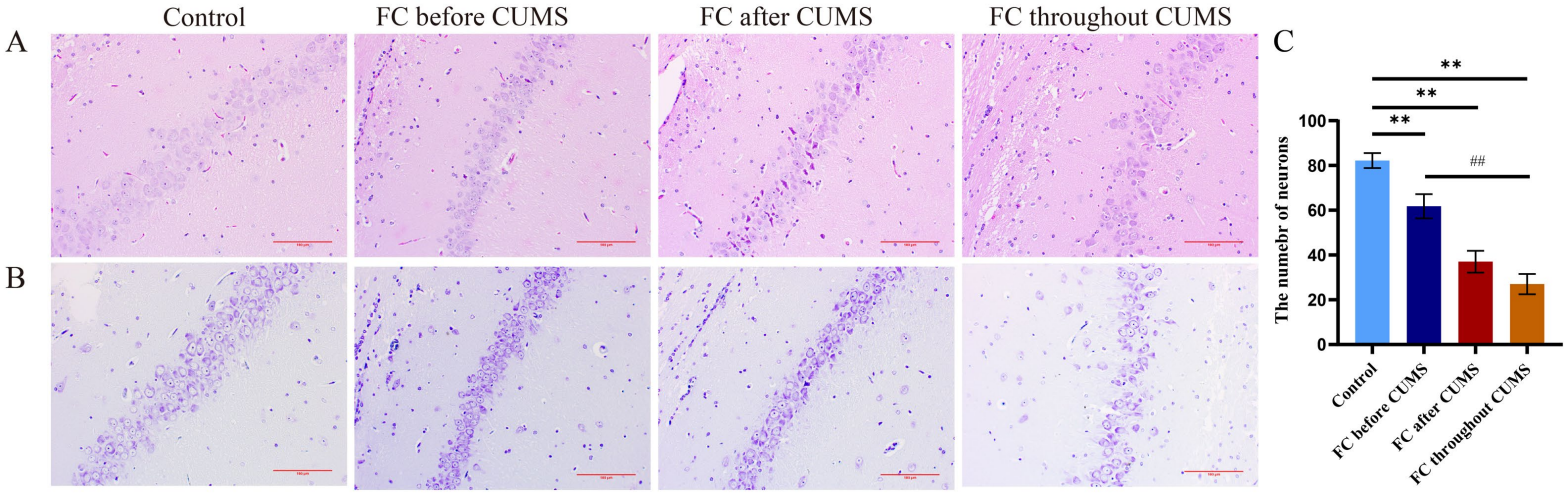
**(A)** HE staining of hippocampal CA1 subregion in each group; **(B)** Nissl staining of hippocampal CA1 subregion in each group (bars, 100 μm); difference from the control group, ^*^p < 0.05, ^**^p < 0.01. Difference from the FC throughout CUMS group, ^#^*p* < 0.05, ^##^*p* < 0.01. Values were expressed as the mean ± SD.

The morphology of neurons in each area of hippocampal tissue in the control group was complete and full, and the neurons were densely arranged and orderly. Compared with the control group, there was a certain degree of damage to the hippocampus of rat in the FC throughout group, which was manifested by unclear nuclear edges, nuclear contraction, pigment agglutination, and disordered cell arrangement. Compared with the FC throughout group, the FC during CUMS group and the FC before/after CUMS groups showed the neurons in a tight and orderly manner, the cell morphology was full, and the apoptosis phenomena, such as nuclear condensation, were significantly reduced (Figures 6A,B). The FC throughout group demonstrated a reduced number of hippocampal CA1 subregion neurons compared to the other groups (Figure 6C).

#### The structure of the mitochondrial

**Figure 7:**
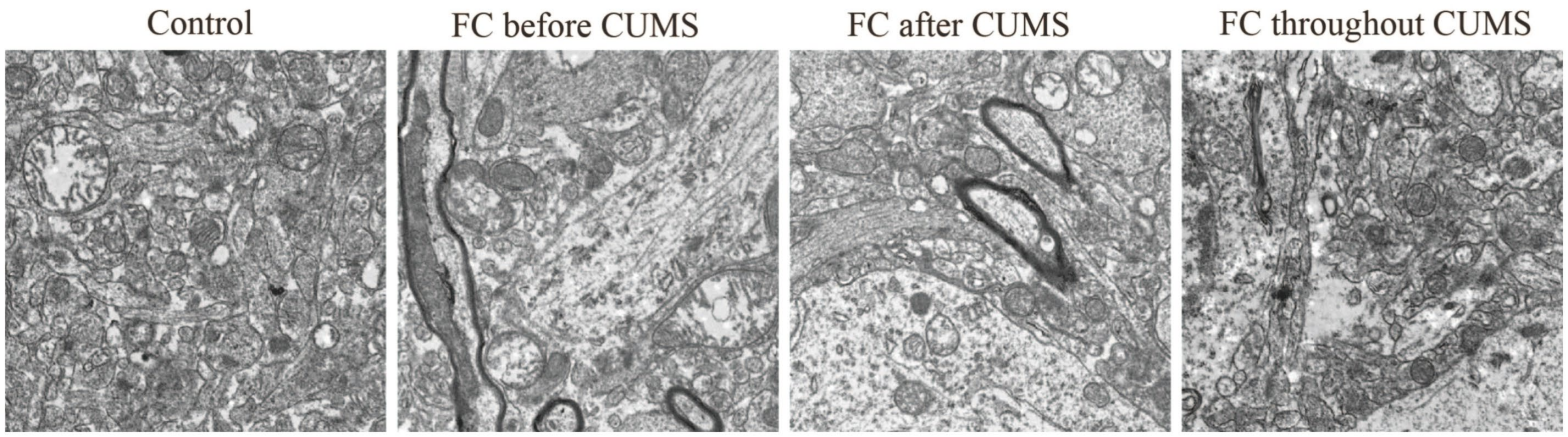
Representative transmission electron microscopy images of the ultrastructure of the hippocampal neurons (bars, 2.0 μm).

Research indicated that oxidative stress played a crucial role in the pathogenesis of depression induced by CUMS, leading to abnormal neuronal apoptosis and neuroinflammation (Bai et al., 2024). Ultrastructure changes of hippocampal neurons in each group were observed by TEM. Hippocampal neurons in the FC throughout group showed mitochondrial membrane rupture and loss of cristae. In contrast, the control group displayed a complete mitochondrial structure. Compared with the FC throughout group, the FC before CUMS and FC after CUMS groups resulted in clearly visible mitochondrial cristae (Figure 7).

#### BDNF and PGP9.5 protein expression in rat hippocampal and Bcl2 and iNOS protein expression in rat Colon

**Figure 8:**
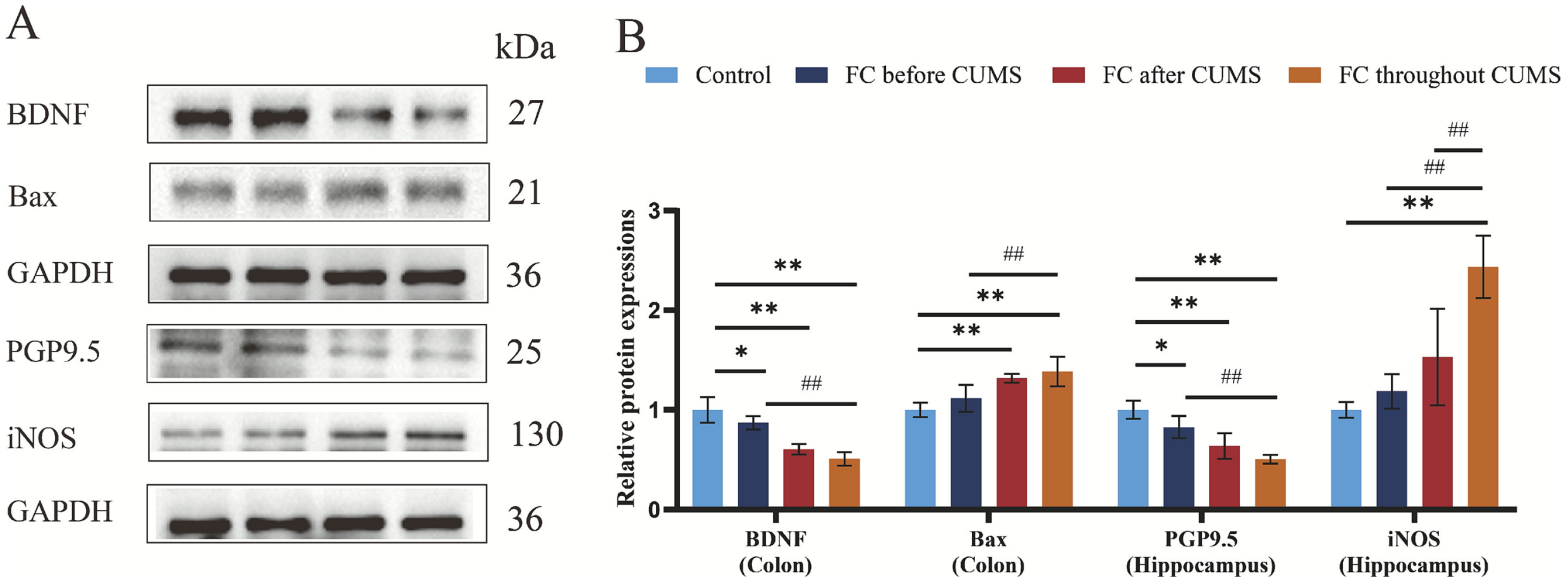
**(A)** WB analysis of the hippocampus neurons and colon protein and **(B)** quantification analysis. Difference from the Control group, *p < 0.05, **p < 0.01. Difference from the FC throughout CUMS group, ##*p* < 0.01. Values were expressed as the mean ± SD.

In terms of the morphology and structure of the enteric nervous system, PGP9.5 demonstrated positive neuronal morphology, which enabled the precise localization of intestinal neurons. The results demonstrated that, in comparison to the control group, the FC throughout group exhibited a decrease in BDNF and PGP9.5, and an increase in Bax, iNOS protein levels (*p* < 0.01), suggesting neuron damage and tissue apoptosis in the compound diphenoxylate, which was combined with CUMS induced rats. The FC before CUMS groups exhibited a marked increase in BDNF and PGP9.5, and a decrease in Bax, iNOS protein expression compared to the FC throughout group (*p* < 0.01) (Figures 8A,B). FC throughout group was worse than other groups. Together, the above data indicated that FC throughout suppressed changes in the levels of hippocampus neurons and promoted colon tissue apoptosis in compound diphenoxylate combined with CUMS induced rats.

## Discussion

This research evaluated the model of FC in rats with depression. Studies found that female FC patients were over twice as likely to suffer from constipation as compared to male FC patients (Zhao et al., 2024). Therefore, we chose female rats to evaluate the model of FC with depression. There was a high comorbidity between constipation and affective disorders like dysphoria and depression (Albiani et al., 2013; Waters et al., 2013), exhibiting symptom exacerbation proportional to disease severity (Moran et al., 2013; Kaplan et al., 1990). There was a growing understanding that the gut-brain axis communicated bidirectionally. Somatic symptoms often accompany dysphoria and depression. Dysphoria can elicit physical symptoms as well (Boven et al., 2011; Larson et al., 2004). On the contrary, somatic symptoms also led to the development of dysphoria and depression (Gerrits et al., 2014). One possible etiologic factor was physical difficulties or limitations caused by somatic symptoms. In addition, common etiologic variables, such as environmental, psychological, and biological factors, may contribute to the development of depression, anxiety disorders, and somatic symptoms (Henningsen et al., 2003). Current research on constipation with depression typically employed animal models where constipation was induced as a measurement of depression indicators. Clinically, patients with constipation and depression often experience depression stemming from chronic constipation. Therefore, selecting models that more closely aligned with clinical realities was particularly crucial.

### Changes in general conditions in FC with depression and anxiety rats

After the end of modeling, the body weight of the FC throughout CUMS group increased slowly. Compared with other groups, the FC throughout group had the worst constipation indicators, such as fecal pellet count, fecal water content, small intestine propulsion rate, and defecating time of first black feces, as well as the behavioral indicators SPT, OFT, and EPM. The constipation and behavioral indicators in the FC before CUMS group was better than in the FC after CUMS group.

Schneider et al. (2023) study found that psychological stress before the onset of colitis had the greatest impact on exacerbation, suggesting psychological stress may precondition the bowel for enhanced inflammation when a colitogenic agent is encountered. This was also consistent with our study, where FC throughout CUMS group of rats had more severe constipation and depression symptoms than the other groups.

### Changes in pathologic indices and the structure of mitochondria in the hippocampal of constipation with depression and anxiety rats

Compared with the FC throughout group, the FC before and after CUMS groups showed the reduction of colon mucosal edema, neatly arranged glands, and reduced inflammatory cell infiltration. The morphology of neurons in each area of hippocampal tissue, compared with the FC throughout group, the FC before and after CUMS groups showed the neurons in a tight and orderly manner, the cell morphology was full, and the apoptosis phenomena, such as nuclear condensation, were significantly reduced.

Ultrastructure changes of hippocampal neurons in each group were observed by TEM. Hippocampal neurons in the FC throughout group showed mitochondrial membrane rupture and loss of cristae. In contrast, the control group displayed a complete mitochondrial structure. Compared with the FC throughout group, FC before CUMS and FC after CUMS groups had clearly visible mitochondrial cristae.

As in the previous CUMS study (He et al., 2023), here we provide evidence in a rat model that revealed animals with FC and CUMS inhibited hippocampal neurogenesis and neuronal proliferation, and these impacts were associated with a growth in depression-like behavior. The FC throughout group showed significant damage in the hippocampus.

### Changes of hippocampal BDNF, Bax, and Colon PGP9.5, iNOS protein expression in the FC with depression and anxiety rats

New neurons in adult brains played a crucial role in the pathogenesis and remission of neuropsychiatric illness (Sahay and Hen, 2007). Neuroinflammation played a significant role in nerve injury and psychiatric disturbances, including depression (Gałecki and Talarowska, 2018; Moghaddam et al., 2023). Long-term chronic inflammation and excessive pressure can contribute to neuroinflammation, cell lesion, and cognitive impairment (Voet et al., 2019), and were found to cause persistent changes in synaptic strength in the hippocampus of stressed rats (Betry et al., 2015). The cholinergic system via acetylcholine regulates behavioral disorders through gut–microbiota–brain signaling, which is also an important neurotransmitter (Wang et al., 2025), and BNDF had the potential to maintain cholinergic receptor function and neurotrophic signaling (Park et al., 2025).

The hippocampus was negatively affected by repeated exposure to stress (Whittle et al., 2014).

Bax is an anti-apoptotic protein, which was required for long-term depression. Research has indicated that depression induced by constipation in mice is accompanied by behavioral alterations and neuronal cell injury (Xu et al., 2018). CUMS induced an increase in the expression of the pro-apoptotic Bax protein, indicating neuronal apoptosis (Wang et al., 2024). Studies show that the increase in Bax expression leads to DNA and chromatin damage (Roostaee et al., 2025). Inhibiting the expression of Bax can reduce the number of impaired hippocampal neurons (Wang et al., 2013).

The gut motility was regulated by a complex collaboration and communication of various ENS related cells, including enteric neurons, Interstitial Cell of Cajal (ICC), and smooth muscles (Al-Shboul, 2013). During the pathogenesis of constipation, significant alterations were detected in the number of enteric neurons and ICC in animal models and human patients. The number of enteric neurons was lower in the submucosal plexus of patients with intractable constipation (Bassotti et al., 2007). Furthermore, a similar decrease pattern was detected in constipated animal models. The levels of NSE and PGP9.5 expression were statistically significantly lower in the middle colon of 16-week old Lep KO mice with the constipation phenotypes (Kim et al., 2020), and Sprague–Dawley rats with loperamide-induced constipation exhibited a decrease in the level of PGP9.5 and BDNF (Kim et al., 2016).

CUMS procedure can induce depressive moods and GI dysfunction simultaneously. BDNF is instrumental in the development and maturation of nerve cells, which is a well-studied neurotrophin known to promote the development and maintenance of serotonergic neurons, thereby influencing 5-HT biosynthesis and availability (Correia et al., 2023). It has been found that BDNF expression is downregulated in depressed patients (Serafini, 2012). Previous studies have shown that the expression of BDNF was decreased in the intestine of mice under CUMS (Sun et al., 2018) and gut motility was decreased in BDNF+/− and constipated mice, with BDNF dose dependently increasing gut motility (Chen et al., 2014). BDNF plays important roles to decrease depression and increase intestinal motility. In our study, compared to the control group, there was a significant reduction in the levels of BDNF and an increase in Bax in the hippocampus of the FC throughout group.

PGP9.5 is a ubiquitin hydrolase that is extensively expressed in the neuronal tissues. PGP9.5 can be used for the characterization of all neurons in ENS, regardless of their actions (Akishima-Fukasawa et al., 2010).

Consistent with previous studies, constipation in rats effectively decreased the number of PGP9.5 positive cells (Wang et al., 2023). Our findings revealed that compared with other groups, the PGP9.5 positive level significantly reduced in the FC and throughout groups. The reduction in PGP9.5 expression during constipation may be due to alterations in neuron numbers, impaired transmission of motor signals, and concurrent decreases in neurotransmitters or obstacles in their synthesis and transmission. These factors collectively led to impaired regulation of intestinal motility, resulting in constipation. Under normal conditions, iNOS was not expressed, but large amounts of iNOS were produced after intestinal damage, resulting in excess NO production, which played a crucial role in the inflammatory response. Decreasing the NO production ameliorated the hyperpermeability of inflamed cells and was linked to the activation of iNOS, which contributed to the inflammatory process (Liu et al., 2021; Guo et al., 2022). Controlling iNOS levels can reduce the NO level and relieve constipation (Tang et al., 2024). In our study, compared to the control group, there was a significant reduction in the levels of PGP9.5 and an increase in iNOS in the colon tissue of the FC throughout group.

Based on our findings, we created a model of constipation with depression as an animal experiment to study the relationship between constipation and depression, most of which were to study depression according to the constipation model. However, most of the FC with depression patients were caused by long-term constipation. Current animal experimental constipation models were established for mostly 2 weeks or 1 month, and did not show a good response to the symptoms of FC with depression patients; Therefore, we combined the constipation and depression model more closely to match the clinical characteristics of the clinical constipation with depression patients.

The study has demonstrated the viability of using rats induced by compound diphenoxylate combined with CUMS model for the study of a successful model for FC with anxiety and depression. There are still many limitations that require further refinement in future studies. First, the specific mechanisms of FC associated with anxiety and depression in the brain–gut axis are still unclear and require further investigation. Second, while many studies have found that females exhibit more severe emotional symptoms, a certain incidence rate of mental disorders still exists among males. Therefore, the experimental results obtained using only female rats cannot be directly extrapolated to male or mixed gender groups. Third, Schneider (Schneider et al., 2023) found that body weight was associated with disease progression, a factor that was not examined in depth in this study. Finally, previous studies have shown that the relative abundance of Proteobacteria, Epsilonbacteraeota, and Verrucomicrobia is higher in diphenoxylate mice, while the relative abundance of Bacteroidetes is higher in healthy and treated groups at the phylum level (Zou et al., 2024). However, we did not validate this further in our study.

## Conclusion

In summary, the present study showed that constipation with CUMS can contribute to depressive behaviors, neuronal lesions in the hippocampus, and colonic tissue damage. Remarkably, all three groups of models showed symptomatic changes in constipation and depression, but they were more severe in the FC throughout group. Therefore, the FC throughout CUMS group was preferably chosen.

## Data Availability

The raw data supporting the conclusions of this article will be made available by the authors, without undue reservation.
